# Energy-Dominated Local Carbon Emissions in Beijing 2007: Inventory and Input-Output Analysis

**DOI:** 10.1100/2012/923183

**Published:** 2012-10-24

**Authors:** Shan Guo, J. B. Liu, Ling Shao, J. S. Li, Y. R. An

**Affiliations:** ^1^State Key Laboratory of Turbulence and Complex Systems, College of Engineering, Peking University, Beijing 100871, China; ^2^Beijing Research Center of Urban System Engineering, Beijing Academy of Science and Technology, Beijing 100089, China

## Abstract

For greenhouse gas (GHG) emissions by Beijing economy 2007, a concrete emission inventory covering carbon dioxide (CO_2_), methane (CH_4_), and nitrous oxide (N_2_O) is presented and associated with an input-output analysis to reveal the local GHG embodiment in final demand and trade without regard to imported emissions. The total direct GHG emissions amount to 1.06*E* + 08 t CO_2_-eq, of which energy-related CO_2_ emissions comprise 90.49%, non-energy-related CO_2_ emissions 6.35%, CH_4_ emissions 2.33%, and N_2_O emissions 0.83%, respectively. In terms of energy-related CO_2_ emissions, the largest source is coal with a percentage of 53.08%, followed by coke with 10.75% and kerosene with 8.44%. Sector 26 (*Construction Industry*) holds the top local emissions embodied in final demand of 1.86*E* + 07 t CO_2_-eq due to its considerable capital, followed by energy-intensive Sectors 27 (*Transport and Storage*) and 14 (*Smelting and Pressing of Ferrous and Nonferrous Metals*). The GHG emissions embodied in Beijing's exports are 4.90*E* + 07 t CO_2_-eq, accounting for 46.01% of the total emissions embodied in final demand. The sound scientific database totally based on local emissions is an important basis to make effective environment and energy policies for local decision makers.

## 1. Introduction

The success of reducing GHG emissions depends greatly on the policies making at urban, domestic and international scales [1]. The international and domestic governments have established general policies (e.g., United Nations Climate Change Conference and China's 12th Five-Year Plan) [2, 3], but the policies enforced at the local level need to be improved by adding more detailed emission pictures within its own territory. Cities contribute 67% to the global GHG emissions from fossil energy use [4], so it is essential and urgent to implement reduction plans at the urban scale. As a result, this paper focuses on local energy inputs and GHG emissions in urban regions to guide environment and energy policies making at the substate level.

Many efforts have been made to calculate environmental emissions at the urban scale [1, 6–8], but most of them about urban carbon emissions just focus on the end-use emissions originated from industrial process, transportation, waste treatment, and so on [9–13], ignoring a deeper understanding of the total emissions in terms of both direct and indirect emissions caused by local commodities' production processes. In fact, all of the commodities consumed in cities lead to GHG emissions during their production processes [8]. For example, the water supply must base on the construction and operation of water works, from which intermediate inputs of steel, concrete, electricity, and so forth are consumed and indirect GHG emissions are produced. As a result, urban planning should consider GHG emissions embodied in commodities used as intermediate inputs to produce products or commodities consumed in cities, not just these obvious direct GHG emissions [5, 14].

To track both direct and indirect effects on embodiments for economies as socioecological systems, input-output analysis (IOA) [15–18] has been applied to analyze embodied GHG emissions [5, 8, 14], energy [19, 20], water resources [21–23], and so forth at urban, domestic, and international scales. Previous input-output studies usually discuss the total emissions (including local and imported emissions) under the assumption that imported commodities have the same embodied intensities as locally produced ones due to the lack of data, which blurs emission sources and responsibility allocation. However, this study highlights local emissions in view of local decision makers without regard to imported emissions. In doing this, based on local GHG emissions inventory, urban policymakers can make low-carbon plans to sustain the sustainable development of cities.

The rate of urbanization will increase from 40% in 2005 to 60% by 2030 in China along with the increasing living standard and the more energy-intensive lifestyle [6]. Taking Beijing as an example, its average annual economy growth rate exceeded 10% while energy consumption growth rate also overtook 6% over the period between 2000 and 2007 [24]. With the rapid development of economy and energy consumption in the near future, more emphasis should be laid on energy consumption and carbon emissions in Beijing. 

With the latest available economic and environmental data, this paper calculates the local GHG emissions by 42 sectors of Beijing in 2007 and further analyzes the local emissions embodied in relevant economic activities based on systems IOA. The rest of this paper is organized as follows. In Section 2, methodological aspects of systems IOA based on the local ecological input-output table and data sources are described.  Section 3 presents the direct GHG emissions inventory and corresponding embodiment analyses for Beijing 2007. Finally, we conclude this study in Section 4 by discussing the results and their implications. 

## 2. Methodology and Data

### 2.1. Local Ecological Input-Output Table

In an attempt to model the local embodiment of natural resources consumption and environmental emissions, a local ecological input-output table extended from the economic input-output table with local economic flows (including local intermediate use and final demand) is compiled as Table 1, integrating direct GHG (including CO_2_, CH_4_, and N_2_O) emissions flows within and across the boundary of the urban economy. 

Taking Beijing as a case, to account the local economic flows, local intermediate use and final demand need to be obtained based on Beijing's competitive economy input-output table. Both intermediate use and final demand can be divided into three parts based on the proportion of local total output, domestic import, and foreign import [25–27]. Therefore, local intermediate input, *z* ^*L*^, can be calculated as

(1) $$\begin{array}{c} z_{ij}^{L}=z_{ij}\left(\frac{x_{i}^{}}{\left(x_{i}^{}+x_{i}^{F}+x_{i}^{D}\right)}\right), \end{array}$$

where *z*_*ij*_ is the total intermediate input from Sector *i* to Sector *j*, *x*_*i*_ is the total output of Sector *i*, *x*_*i*_^*F*^ is the foreign imported economic flow of Sector *i*, and *x*_*i*_^*D*^ is the domestic imported economic flow of Sector *i*. While final demand of Sector *i* from local output,*f*_*i*_^*L*^, is expressed as

(2) $$\begin{array}{c} f_{i}^{L}=f_{i}\left(\frac{x_{i}^{}}{\left(x_{i}^{}+x_{i}^{F}+x_{i}^{D}\right)}\right), \end{array}$$

where *f*_*i*_ is the total final demand of Sector *i*.

### 2.2. Algorithm

**Figure 1:**
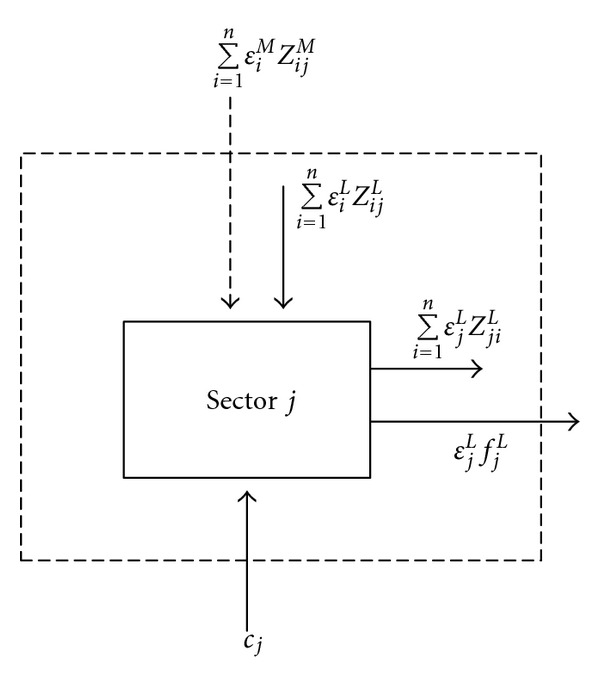
Embodied GHG flows for a typical sector in an urban economy (carbon flows introduced by imported commodities from other domestic and foreign regions ∑_*i*=1_^*n*^ *ε*_*i*_^*M*^ *z*_*ij*_^*M*^ are not considered based on local emissions).

From the perspective of local decision makers, this study focuses only on carbon flows coming from the urban system without taking into account carbon flows coming from the international and domestic systems. The embodied carbon flows for a typical sector in an urban economy based on local emissions can be described as Figure 1, including local and imported intra- and inter- sectoral carbon flows (*ε*_*i*_^*L*^ is the local embodied intensity of products from Sector *i*, *z*_*ij*_^*L*^ is the monetary value of local intermediate inputs from Sector *i* to Sector *j*, *ε*_*i*_^*M*^ is the imported embodied intensity of products from Sector *i*, *z*_*ij*_^*M*^ is the monetary value of imported intermediate inputs from Sector *i* to Sector *j*, *ε*_*j*_^*L*^ is the local embodied intensity of products from Sector *j*, and *z*_*ji*_^*L*^ is the monetary value of local intermediate inputs from Sector *j* to Sector *i*), carbon flows embodied in final demand (*f*_*j*_^*L*^ denotes the final demand of Sector *j* from local outputs), and net environmental inputs flows (*c*__*j*__ is the amount of direct GHG emissions).

Based on Figure 1, the sectoral biophysical balance requires that

(3) $$\begin{array}{c} \varepsilon_{j}^{L}x_{j}=\sum_{i=1}^{n}\varepsilon_{i}^{L}z_{ij}^{L}+\sum_{i=1}^{n}\varepsilon_{i}^{M}z_{ij}^{M}+c_{j}, \end{array}$$

where *x*_*j*_ is the monetary value of total outputs of Sector *j*.

To calculate local embodied emissions in this paper, emissions introduced by imported commodities from other domestic and foreign regions are not concerned. Then, rewrite the physical balance equation as

(4) $$\begin{array}{c} \varepsilon_{j}^{L}x_{j}=\sum_{i=1}^{n}\varepsilon_{i}^{L}z_{ij}^{L}+c_{j}. \end{array}$$

Then an aggregate matrix equation can be induced as:

(5) $$\begin{array}{c} E^{L}X=E^{L}Z^{L}+C, \end{array}$$

in which *E* ^*L*^ = [*ε*_*j*_^*L*^]_1×*n*_,  *Z* ^*L*^ = [*z*_*ij*_^*L*^]_*n*×*n*_,  *C* = [*c*_*j*_]_1×*n*_, and *X* = [*x*_*ij*_]_*n*×*n*_, where *i*,  *j* ∈ (1,2,…, *n*),  *x*_*ij*_ = *x*_*j*_(*i* = *j*), and *x*_*ij*_ = 0  (*i* ≠ *j*).

Therefore, with direct GHG emissions matrix *C*, local intermediate input matrix *Z* ^*L*^, and total outputs matrix *X* properly given, the embodied GHG emissions intensity matrix *E* ^*L*^ can be calculated as

(6) $$\begin{array}{c} E^{L}=C{\left(X-Z^{L}\right)}^{-1}. \end{array}$$

Though very similar to the conventional formula based on the widely assumed equal embodiment intensity for both the local and import products, the above formal equation for local embodiment intensity has different implications. It reflects the embodied intensity induced by local direct emissions but ignores that induced by imported emissions. Therefore, local direct and indirect emissions can be clearly demonstrated.

Evidently, the GHG emissions embodied in final demand activities, denoted by EEFD [5, 27], can be calculated as the product of embodied intensity and corresponding final demand volume from Sector *j*, as

(7) $$\begin{array}{c} \text{EEF}{\text{D}}_{j}=\varepsilon_{j}^{L}f_{j}^{L}. \end{array}$$

Emission embodied in trade is a useful indicator to reveal transferring carbon emissions. Focusing on local emissions, emissions embodied in trade include emissions embodied in exports but exclude emissions embodied in imports. Combining GHG emissions from other domestic and foreign regions, GHG emissions embodied in exports (EEE_*j*_), including emissions embodied in exports to other domestic regions (EEE__*j*__^*D*^) and exports to foreign regions (EEE__*j*__^*F*^), can be expressed as

(8) $$\begin{array}{c} \text{EE}{\text{E}}_{j}=\text{EE}{\text{E}}_{j}^{D}+\text{EE}{\text{E}}_{j}^{F}=\varepsilon_{j}^{L}e_{j}^{D}+\varepsilon_{j}^{L}e_{j}^{F}, \end{array}$$

where *e*_*j*_^*D*^ and *e*_*j*_^*F*^ denote the export to other domestic regions and export to foreign regions of Sector *j*.

### 2.3. Data Sources

Most relevant environmental resources and economic data are adopted or derived from the recently issued official statistical yearbooks, such as Beijing Statistical Yearbook [28], China Agriculture Yearbook [29], China Energy Statistical Yearbook [30], China Environment Yearbook [31], China Industry Economics Statistical Yearbook [32], and China Statistical Yearbook for Regional Economy [33].

In this paper, all the three main GHG emissions of CO_2_, CH_4_, and N_2_O are taken into consideration. The calculation of energy-related CO_2_ emissions is based on a previous study [5], and the energy consumption data sources are from BSY and CESY by utilizing the default emission factors of IPCC [34]. For CO_2_ emissions from industrial processes, the data of industrial products can be found in BSY, CIESY, and other sources. And corresponding emission factors are also adopted from IPCC combined with Chen and Zhang [14]. As to CH_4_ and N_2_O, the data from different emission sources are derived from BSY, CAY, CESY, CEY, CIESY, and other databases. Since some specific emission factors need to suit the Chinese situation, this paper adopts the emission factors from Chen and Zhang [14].

Obtained from the most recently available Beijing Bureau of Statistics, the Beijing's economic input-output table 2007 is adopted. In this table, the Beijing economy is divided into 42 sectors, including 1 sector for the first industry, 25 sectors for the second industry, and 16 sectors for the third industry, as listed in Table 2. The economic flows of input-output table are based on producer prices in 2007 with a unit of ten thousand Chinese Yuan.

## 3. Results

### 3.1. Direct Emissions

#### 3.1.1. Carbon Dioxide

**Figure 2:**
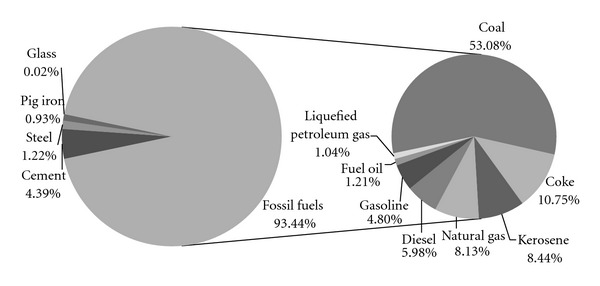
The components of direct CO_2_ emissions by source.

The total direct CO_2_ emissions amount to 1.01*E* + 08 t. Guo et al. [5] provide a detailed inventory of energy-related direct CO_2_ emissions by fuel consumption in Beijing. As the largest emission source, fuel combustion contributes to 93.44% of total. Among the emissions from fuel combustion, the largest source is coal with a percentage of 53.08%, followed by coke with 10.75% and kerosene with 8.44% (see Figure 2). 

Compared with fuel consumption, industrial processes are only responsible for 6.64*E* + 06 t CO_2_ emissions (6.56%), of which 4.44*E* + 02 t is contributed by manufacturing of cement (4.39%), 1.24*E* + 02 t is by smelting and pressing of steel (1.22%), 9.37*E* + 01 t is by smelting and pressing of pig iron (0.93%) and 2.35*E* + 00 t is by manufacturing of glass (0.02%).

#### 3.1.2. Methane

The main sources of CH_4_ emission include agricultural activities (enteric fermentation, manure management, and field burning of plant residues), fugitive emissions (coal mining, oil and natural gas leakage), fossil fuel consumption, and waste (municipal solid waste, industrial wastewater, and domestic sewage) [14]. From the calculation, it is obtained that the total CH_4_ emissions amount to 1.18*E* + 01 t. As the most important source of methane emissions, the solid waste accounts for 45.48% of total, followed by enteric fermentation and coal mining with 17.79% and 13.81%, respectively. However, fossil fuel consumption only accounts for 1.43% of total, as shown in Table 3.

As the main source, agriculture activities cause 2.50*E* + 00 t CH_4_, of which emissions from enteric fermentation amount to 2.10*E* + 00 t as 17.79% of total, followed by manure management and field burning of agricultural residues with the fractions of 2.55% and 0.86%. 

Fugitive CH_4_ emission sources in Beijing include coal mining with oil and natural gas systems, of which the CH_4_ emissions are 1.63*E* + 00 t and 1.14*E* + 00 t, respectively. The total fugitive CH_4_ emissions are 2.77*E* + 00 t, accounting for 23.46% of total.

With expansion of urban population, urban waste problems become increasingly severe. Among the CH_4_ emissions of waste, emissions from municipal solid waste (5.37*E* + 00 t, 45.48% of total) play a main role compared to industrial waste water (4.90*E* − 01 t, 4.15% of total) and domestic sewage (5.03*E* − 01 t, 4.26% of total). 

#### 3.1.3. Nitrous Oxide

Direct N_2_O emissions in Beijing from main sources like agricultural activities (manure management, cropland, and field burning of agricultural residues) and fuel combustion (see Table 4) are estimated in this paper. The total emissions of N_2_O from all sources amount to 2.84*E* − 01 t, which are far less than those of CO_2_ and CH_4_ by mass, but the global warming potential of N_2_O is the greatest among these three greenhouse gases (CO_2_ : CH_4_ : N_2_O = 1 : 21 : 310).

Considerable N_2_O emissions are caused by agricultural activities (58.09%) in Beijing. Cropland contributes the most to N_2_O emissions from annual synthetic fertilizer (29.23%), followed by manure management (28.20%) and field burning of agricultural residues (0.67%). Besides, the N_2_O emissions from fuel combustion are 1.19*E* − 01 t, 41.91% of total in Beijing. Since Beijing has no nitric acid and adipic acid products, N_2_O emission coming from industrial processes can be ignored.

As to N_2_O emissions by sector, agriculture sector contributes to the largest emissions (1.67*E* − 01 t, 58.77% of total), which is due to massive N_2_O emissions from cropland and manure management, while other sectors perform poorly in N_2_O emissions. Therefore, effective management and control of agriculture activities is an effective way to reduce N_2_O emissions.

#### 3.1.4. Total Emissions

**Figure 3:**
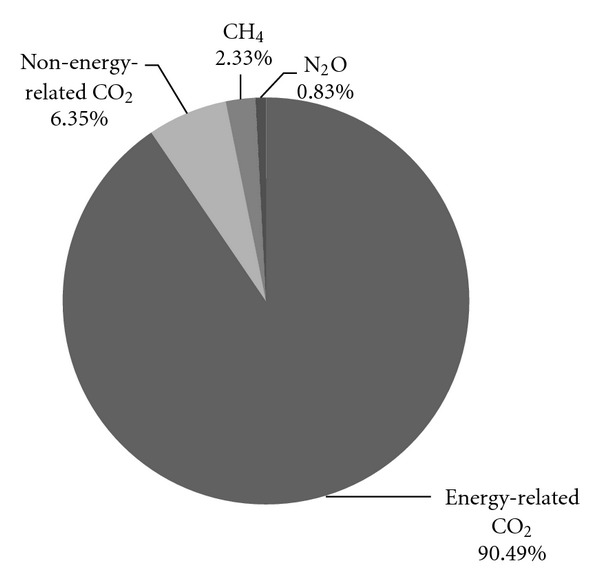
The components of GHG emissions.

The total direct GHG emissions amount to 1.06*E* + 08 t CO_2_-eq in Beijing 2007 by the commonly referred IPCC global warming potentials, of which energy-related CO_2_ contributes to 9.45*E* + 07 t CO_2_-eq (90.49% of total), non-energy-related CO_2_  6.64*E* + 06 t CO_2_-eq (6.35% of total), CH_4_  2.48*E* + 06 t CO_2_-eq (2.33% of total), and N_2_O 8.81*E* + 05 t CO_2_-eq (0.83% of total) as shown in Figure 3. 

With all the categories mentioned above, total direct GHG emissions are presented in Table 5, of which Sector 23 (*Electric Power/Steam and Hot Water Production and Supply*) contributes to the largest share of GHG emissions, which amount to 2.79*E* + 07 t CO_2_-eq (26.20% of total), followed by Sectors 14 (*Smelting and Pressing of Ferrous and Nonferrous Metals*), 27* (Transport and Storage),* and 13 *(Nonmetal Mineral Products)* with 2.08*E* + 07 t CO_2_-eq (19.54% of total), 1.43*E* + 07 t CO_2_-eq (13.40% of total), and 1.03*E* + 07 t CO_2_-eq (9.68% of total), respectively. Sector 23 is the energy conversion sector, while Sectors 14, 27, and 13 are all energy-intensive sectors. A host of GHG emissions are derived from aluminum production in Sector 14, and Sector 13 emits considerable GHG due to the production of nonmetallic mineral products including concrete and glass besides energy-related emissions. 

With comparison of GHG emissions shown in Table 5, it is noted that CH_4_ and N_2_O emissions are tiny, excluding those in Sectors 1 *(Agriculture)* and 2 *(Coal Mining and Dressing)* attributed to agricultural activities and fugitive emissions. Direct CH_4_ emissions of Sectors 1 and 2 amount to 5.26*E* + 05 and 3.42*E* + 05 t CO_2_-eq, accounting for 21.22% and 13.80% of the total CH_4_ emissions. Sector 1 is the leading N_2_O emission sector with 5.18*E* + 05 t CO_2_-eq, accounting for 81.27% of the total N_2_O emissions.

### 3.2. Embodied Emissions

#### 3.2.1. Embodied Emission Intensity

**Figure 4:**
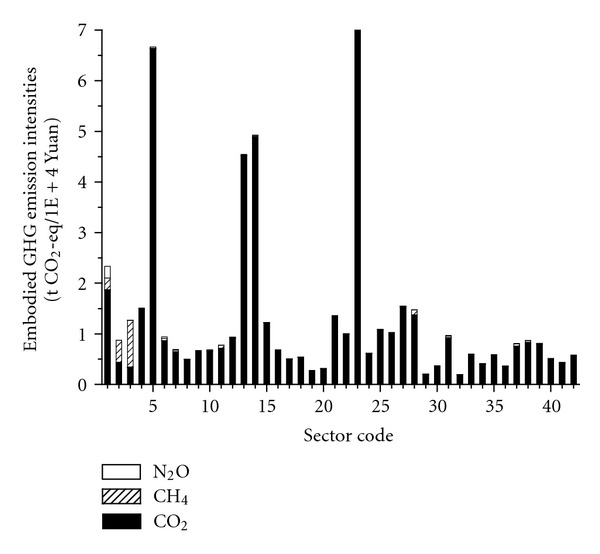
Embodied GHG emission intensities of 42 sectors.

As presented in Figure 4 for the local embodied GHG emission intensities of 42 sectors in Beijing 2007 based on (6) and Table 5, Sector 23 (*Electric Power/Steam and Hot Water Production and Supply*) has the largest intensity of 7.06 t CO_2_-eq/1*E* + 4 Yuan, followed by Sectors 5 (*Nonmetal and Other Minerals Mining and Dressing*), 14 (*Smelting and Pressing of Ferrous and Nonferrous Metals*), and 13 (*Nonmetal Mineral Products*) with intensities of 6.67, 4.93, and 4.55 t CO_2_-eq/1*E* + 4 Yuan, respectively. More evidently, these high-intensity sectors are all characterized by remarkable direct emissions.

According to the emission type, embodied GHG emission intensities of most industries are dominated by the embodied CO_2_ emission industries, except for Sectors 2 (*Coal Mining and Dressing*) and 3 (*Petroleum and Natural Gas Extraction*). The shares of CH_4_ emission intensities of Sectors 1 (*Agriculture*), 2 (*Coal Mining and Dressing*), and 3 (*Petroleum and Natural Gas Extraction*) are especially high. The proportion of N_2_O emissions intensities for most sectors is small except for Sector 1 (*Agriculture*) since agriculture activities are the main sources of N_2_O emissions.

#### 3.2.2. Emissions Embodied in Final Demand

**Figure 5:**
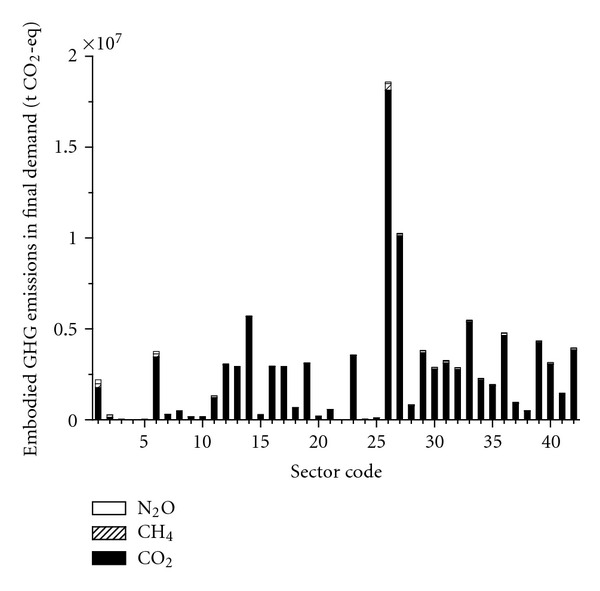
Emissions embodied in final demand.

As shown in Figure 5, the final demand activities of Beijing in terms of embodied GHG emissions are presented according to (7). The largest GHG-emission sector is Sector 26 (*Construction Industry*) with 1.86*E* + 07 t CO_2_-eq due to its considerable fixed capital. With the strong growth of construction in Beijing, lots of direct and indirect inputs (e.g., cement, metal, and energy) are produced during these construction activities, which lead to a great deal of carbon emissions. Sectors 27 (*Transport and Storage*) and 14 (*Smelting and Pressing of Ferrous and Nonferrous Metals*) provide the second and third largest emissions of 1.03*E* + 07 and 5.72*E* + 06 t CO_2_-eq, mainly attributed to their substantial exports to foreign regions and other domestic regions, respectively. Besides, GHG emissions of Sector 27 are also introduced by massive government consumption and urban household consumption with rising traffic consumption level. Most sectors have prominent peaks on CO_2_ emissions; Sectors 1 (*Agriculture*) and 6 (*Food Processing, Food Production, Beverage Production, *and *Tobacco Processing*) are also with massive CH_4_ emissions due to agriculture activities, while Sector 26 (*Construction Industry*) are due to high energy usage. Especially for Sector 2 (*Coal Mining and Dressing*), CH_4_ emissions contribute to 49.37% of the total due to this particular industrial process in Beijing.

**Figure 6:**
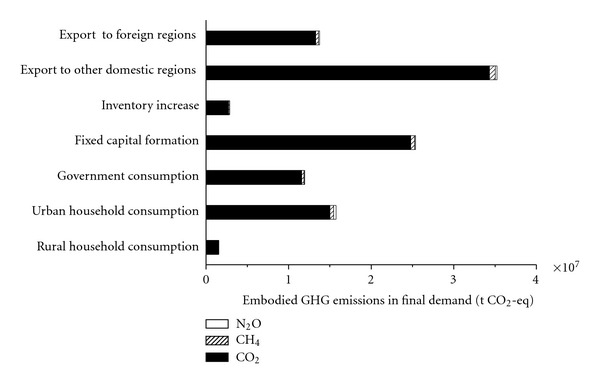
The components of embodied GHG emissions by final demand category.

Regarding the seven final demand categories (see Figure 6), emissions embodied in exports to other domestic regions have the largest value of 3.52*E* + 07 t CO_2_-eq, accounting for 33.10% of total. Besides, GHG emissions embodied in fixed capital formation are responsible for 23.83% of total due to intensive investment with the urban construction boom in Beijing. Emissions embodied in rural household consumption (1.55*E* + 06 t CO_2_-eq, 1.46% of total) are just 9.86% of those in urban household consumption (1.57*E* + 07 t CO_2_-eq, 14.78% of total). Emissions embodied in government consumption (1.19*E* + 07 t CO_2_-eq, 11.22% of total) are 30.89% less than those in household consumption (urban and rural). 

#### 3.2.3. Emissions Embodied in Exports

**Figure 7:**
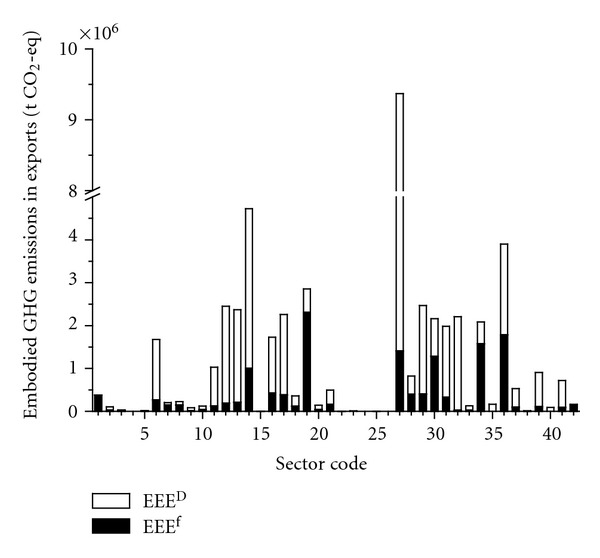
Emissions embodied in exports.

Since local emissions embodied in trade only focus on emissions induced by local direct emissions but do not take imports into account, this paper just studies the exports to foreign regions and other domestic regions excluding imports. The distribution of embodied emissions from the exports in 42 sectors is presented in Figure 7. The GHG emission embodied in Beijing's exports is 4.90*E* + 07 t CO_2_-eq, accounting for 46.01% of the total emissions in final use. The total EEE_  _^*D*^ (3.52*E* + 07 t CO_2_-eq) are 2.56 times larger than the total EEE_  _^*F*^ (1.37*E* + 07 t CO_2_-eq) for Beijing. The largest exporting sector is Sector 27 (*Transport and Storage, *9.37*E* + 06 t CO_2_-eq, 19.12% of total), followed by Sectors 14 (*Smelting and Pressing of Ferrous and Nonferrous Metals, *4.72*E* + 06 t CO_2_-eq, 9.64% of total), 36 (*Polytechnic Service, *3.90*E* + 06 t CO_2_-eq, 7.96% of total), and 19 (*Electronic and Telecommunications Equipment, *1.85*E* + 06 t CO_2_-eq, 5.82% of total). As a whole, most sectors have the larger EEE_  _^*D*^ except for some large foreign trade export sectors, for example, Sectors 1, 3, 7, 8, 19, 30, 34, and 42 with larger EEE_  _^*F*^. 

## 4. Concluding Remarks

This paper provides a systematic and detailed calculation on the embodiment of local GHG emissions at urban scale through the extended economic input-output analysis with the case study of Beijing 2007. As a result, a local direct GHG emissions inventory and corresponding embodiment analyses are assessed.

The total direct GHG emissions amount to 1.06*E* + 08 t CO_2_-eq in Beijing. For the total emissions structure, energy-related CO_2_ emissions comprise 90.49%, non-energy-related CO_2_ emissions 6.35%, CH_4_ emissions 2.33%, and N_2_O emissions 0.83%. Among the emissions from fuel combustion, the largest source is coal with a percentage of 53.08%, followed by coke with 10.75% and kerosene with 8.44%. Sector 23 (*Electric Power/Steam and Hot Water Production and Supply*) is the largest direct emissions sector for the Beijing economy in 2007, followed by energy-intensive Sectors 14 (*Smelting and Pressing of Ferrous and Nonferrous Metals*), 27* (Transport and Storage),* and 13 *(Nonmetal Mineral Products)*. 

For the final demand of Beijing in terms of embodied CO_2_ emissions, Sector 26 (*Construction Industry*) provides the largest emissions of 1.86*E* + 07 t CO_2_-eq due to its considerable capital during the concerned year. Sectors 27 (*Transport and Storage*) and 14 (*Smelting and Pressing of Ferrous and Nonferrous Metals*) provide the second and third largest emissions of 1.03*E* + 07 and 5.72*E* + 06 t CO_2_-eq. 

The GHG emissions embodied in Beijing's exports are 4.90*E* + 07 t CO_2_-eq, accounting for 46.01% of the total emissions in final demand. The total EEE_  _^*D*^ (3.52*E* + 07 t CO_2_-eq) are 2.56 times larger than the total EEE_  _^*F*^ (1.37*E* + 07 t CO_2_-eq) for Beijing. The largest exporting sector is Sector 27 (*Transport and Storage)*, followed by Sectors 14 (*Smelting and Pressing of Ferrous and Nonferrous Metals*), 36 (*Polytechnic Service*), and 19 (*Electronic and Telecommunications Equipment*). 

Resulted embodied local GHG intensities for sectors indicate the average amount of local emissions embedded in one economic unit of local product, which provide sound scientific data for local policy makers to adjust industrial structure and energy consumption structure in order to relieve global climate change. From the perspective of local decision makers, this study is an important basis when local environment and energy policies are making. 

Expansion of industrial scale has been the main driving factor of energy consumption and carbon emissions. While the change of industrial structure and maximize energy efficiency are effective measures to conserve energy and reduce emissions. Specific measures are as follows: (1) In terms of energy efficiency, local government continuously eliminates high-energy-consumption industries and backward production capacity. In the meantime, they should in favor of high and advanced production technology to maximize energy efficiency, especially for some high-energy-consumption or high-resource-consumption industries, such as Sectors 23 (*Electric Power/Steam and Hot Water Production and Supply*), 14 (*Smelting and Pressing of Ferrous and Nonferrous Metals*), etc. (2) Industrial structural change makes great impact on carbon emissions structure. Beijing has made great efforts for industrial structural change, for example, Beijing is greatly developing the tertiary industries and reducing the proportion of primary industries and secondary industries. However, detailed industrial structure should be adjusted based on the carbon consuming responsibilities. 

## Figures and Tables

**Table 1:** The local ecological input-output table (*C* is the direct GHG emissions matrix).

		Output										
Input		Intermediate use				Final demand						
		Sector 1	Sector 2	…	Sector *n *	Household consumption (rural)	Household consumption (urban)	Government consumption	Fixed capital formation	Inventory increase	Export to other domestic regions	Export to foreign regions
Local intermediate inputs	Sector 1											
	Sector 2			*Z* ^*L*^					*F* ^*L*^			
	…											
	Sector *n *											

Net environmental inputs	CO_2_											
	CH_4_		*C*									
	N_2_O											

**Table 2:** Sectors for Beijing's economic input-output table 2007 [5].

Code	Sector
1	Farming, Forestry, Animal Husbandry, Fishery, and Water Conservancy (Agriculture)
2	Coal Mining and Dressing
3	Petroleum and Natural Gas Extraction
4	Ferrous and Nonferrous Metals Mining and Dressing
5	Nonmetal and Other Minerals Mining and Dressing
6	Food Processing, Food Production, Beverage Production, and Tobacco Processing
7	Textile Industry
8	Garments and Other Fiber Products, Leather, Furs, and Down and Related Products
9	Timber Processing, Bamboo, Cane, Palm and Straw Products, and Furniture Manufacturing
10	Papermaking and Paper Products, Printing and Record Medium Reproduction, and Cultural, Educational, and Sports Articles
11	Petroleum Processing and Coking, Gas Production and Supply
12	Raw Chemical Materials and Chemical Products, Medical and Pharmaceutical Products, Chemical Fiber, Rubber Products, and Plastic Products (Chemical Products Related Industry)
13	Nonmetal Mineral Products
14	Smelting and Pressing of Ferrous and Nonferrous Metals
15	Metal Products
16	Ordinary Machinery, Equipment for Special Purpose
17	Transportation Equipment
18	Electric Equipment and Machinery
19	Electronic and Telecommunications Equipment
20	Instruments, Meters Cultural and Office Machinery
21	Manufacture of Artwork and Other Manufactures
22	Waste
23	Electric Power/Steam and Hot Water Production and Supply
24	Gas Production and Supply Industry
*25 *	Water Production and Supply Industry
26	Construction Industry
27	Transport and Storage
28	Post
29	Information Transmission, Computer Services and Software
30	Wholesale, Retail Trade
31	Hotels, Catering Service
32	Financial Industry
33	Real Estate
34	Leasing and Commercial Services
35	Research and Experimental Development
36	Polytechnic Services
37	Water conservancy, Environment and Public Facilities Management
38	Service to Households and Other Service
39	Education
40	Health, Social Security, and Social Welfare
41	Culture, Sports, and Entertainment
42	Public Management and Social Organization

**Table 3:** Anthropogenic methane emissions by source.

Item	CH_4_ emission (t)	Fraction
(1) Agriculture activities	2.50*E* + 00	21.21%
Enteric fermentation	2.10*E* + 00	17.79%
Manure management	3.01*E* − 01	2.55%
Field burning of agricultural residues	1.02*E* − 01	0.86%
(2) Fugitive emissions	2.77*E* + 00	23.46%
Coal mining	1.63*E* + 00	13.81%
Oil and natural gas systems	1.14*E* + 00	9.66%
(3) Fossil fuel combustion	1.69*E* − 01	1.43%
(4) Waste	6.36*E* + 00	53.90%
Municipal solid waste	5.37*E* + 00	45.48%
Industrial waste water	4.90*E* − 01	4.15%
Domestic sewage	5.03*E* − 01	4.26%

(5) Total	1.18*E* + 01	100.00%

**Table 4:** Anthropogenic nitrous oxide emissions by source.

Item	N_2_O emission (t)	Fraction
(1) Fossil fuel combustion	1.19*E* − 01	41.91%
(2) Agriculture activities	1.65*E* − 01	58.09%
Manure management	8.01*E* − 02	28.20%
Cropland	8.31*E* − 02	29.23%
Field burning of agricultural residues	1.90*E* − 03	0.67%

(3) Total	2.84*E* − 01	100.00%

**Table 5:** Direct GHG emissions by type and sector.

Sector code	CO_2_ (t)	CH_4_ (t CO_2_-eq)	N_2_O (t CO_2_-eq)	Total GHGs (t CO_2_-eq)	Fraction
1	3.44*E* + 06	5.26*E* + 05	5.18*E* + 05	4.48*E* + 06	4.21%
2	5.96*E* + 04	3.42*E* + 05	2.70*E* + 02	4.02*E* + 05	0.38%
3	3.81*E* + 04	2.39*E* + 05	9.58*E* + 01	2.78*E* + 05	0.26%
4	9.81*E* + 04	4.19*E* + 01	4.06*E* + 02	9.86*E* + 04	0.09%
5	2.16*E* + 05	8.11*E* + 01	8.71*E* + 02	2.17*E* + 05	0.20%
6	1.58*E* + 06	4.69*E* + 04	7.27*E* + 03	1.64*E* + 06	1.54%
7	2.39*E* + 05	1.94*E* + 04	1.10*E* + 03	2.60*E* + 05	0.24%
8	3.24*E* + 05	9.54*E* + 01	1.48*E* + 03	3.25*E* + 05	0.31%
9	9.17*E* + 04	3.90*E* + 01	3.53*E* + 02	9.21*E* + 04	0.09%
10	4.66*E* + 05	2.97*E* + 04	1.92*E* + 03	4.97*E* + 05	0.47%
11	1.19*E* + 06	4.89*E* + 02	2.71*E* + 03	1.19*E* + 06	1.12%
12	2.99*E* + 06	1.01*E* + 04	1.33*E* + 04	3.02*E* + 06	2.83%
13	1.03*E* + 07	1.68*E* + 03	2.63*E* + 04	1.03*E* + 07	9.68%
14	2.07*E* + 07	3.96*E* + 03	8.43*E* + 04	2.08*E* + 07	19.54%
15	2.27*E* + 05	9.59*E* + 01	8.55*E* + 02	2.28*E* + 05	0.21%
16	8.19*E* + 05	2.63*E* + 02	3.50*E* + 03	8.23*E* + 05	0.77%
17	9.11*E* + 05	2.88*E* + 02	3.78*E* + 03	9.15*E* + 05	0.86%
18	1.22*E* + 05	5.17*E* + 01	4.17*E* + 02	1.23*E* + 05	0.12%
19	1.32*E* + 05	6.12*E* + 01	1.99*E* + 02	1.32*E* + 05	0.12%
20	3.73*E* + 04	2.02*E* + 01	1.19*E* + 02	3.74*E* + 04	0.04%
21	2.09*E* + 05	5.54*E* + 01	9.75*E* + 02	2.10*E* + 05	0.20%
22	8.67*E* + 03	4.47*E* + 00	3.31*E* + 01	8.71*E* + 03	0.01%
23	2.78*E* + 07	6.74*E* + 03	1.28*E* + 05	2.79*E* + 07	26.20%
24	1.18*E* + 05	6.06*E* + 01	1.34*E* + 02	1.18*E* + 05	0.11%
25	1.70*E* + 04	8.34*E* + 00	4.84*E* + 01	1.71*E* + 04	0.02%
26	1.27*E* + 06	3.04*E* + 05	3.78*E* + 03	1.57*E* + 06	1.48%
27	1.42*E* + 07	8.00*E* + 04	3.58*E* + 04	1.43*E* + 07	13.40%
28	7.74*E* + 05	6.91*E* + 04	1.96*E* + 03	8.45*E* + 05	0.79%
29	1.83*E* + 05	6.85*E* + 04	4.70*E* + 02	2.52*E* + 05	0.24%
30	1.81*E* + 06	1.07*E* + 05	5.04*E* + 03	1.93*E* + 06	1.81%
31	2.73*E* + 06	1.06*E* + 05	5.20*E* + 03	2.84*E* + 06	2.67%
32	1.47*E* + 05	4.68*E* + 04	4.24*E* + 02	1.94*E* + 05	0.18%
33	3.54*E* + 06	4.78*E* + 04	1.18*E* + 04	3.60*E* + 06	3.38%
34	1.20*E* + 06	4.73*E* + 04	4.02*E* + 03	1.25*E* + 06	1.17%
35	4.15*E* + 05	4.69*E* + 04	1.33*E* + 03	4.63*E* + 05	0.43%
36	4.15*E* + 05	4.69*E* + 04	1.33*E* + 03	4.63*E* + 05	0.43%
37	4.26*E* + 05	4.69*E* + 04	1.48*E* + 03	4.74*E* + 05	0.45%
38	8.28*E* + 05	4.70*E* + 04	2.89*E* + 03	8.78*E* + 05	0.82%
39	1.63*E* + 06	4.73*E* + 04	4.85*E* + 03	1.69*E* + 06	1.58%
40	5.05*E* + 05	4.69*E* + 04	1.47*E* + 03	5.53*E* + 05	0.52%
41	2.83*E* + 05	4.69*E* + 04	7.52*E* + 02	3.30*E* + 05	0.31%
42	7.19*E* + 05	4.71*E* + 04	2.32*E* + 03	7.68*E* + 05	0.72%

Total	1.03*E* + 08	2.48*E* + 06	8.81*E* + 05	1.06*E* + 08	100.00%
